# Developmental basis of male nipple loss and retention in mammals

**DOI:** 10.21203/rs.3.rs-7697632/v1

**Published:** 2025-10-24

**Authors:** Zhengui Zheng, Shanshan Wang, Amber Cooke

**Affiliations:** Southern illinois University Carbondale; Southern Illinois University Carbondale; Monroe Carell Jr. Children’s Hospital at Vanderbilt

**Keywords:** Mammary gland development, male nipple loss, nipple retention, guinea pig and mouse

## Abstract

Most male mammals, including humans, have nipples, but a few male species, including mice, do not. It is currently believed that testosterone produced by the fetal testes during sexual differentiation causes nipple atrophy in male mice. However, since all male mammals produce testosterone during sexual differentiation, how nipples are retained in most male mammals, including humans, remains unknown. This study compared mammary gland development and hormonal regulation between guinea pigs and mice and found that androgen receptor (AR) is exclusively present in the developing mammary glands of mice during sexual differentiation. In mice, testosterone binds to AR, activating androgen-responsive genes and inducing apoptosis and autophagy in epithelial cells of the nipple bud, leading to nipple atrophy. During guinea pig embryonic development, the absence of the AR inactivates AR pathway genes and activates Wnt pathway genes, promoting cell proliferation and inhibiting programmed cell death, thereby inducing nipple formation.

## Introduction

The mammary gland (MG), which distinguishes mammals from all other animals, functions to produce and secrete milk in order to nourish offspring. The fundamental structural and functional properties of the MG are conserved across mammalian species^1^. Except for fruit bats, MGs of most male mammals do not perform lactation, and the majority of male mammals don’t have fully developed and functional MGs, but they do have nipples^1,2^. Males of only a few species, such as rat (*Rattus norvegicus*), mouse (*Mus musculus*) and horse (*Equus caballus*) don’t have nipples^3–5^. The main function of MG is to nourish offspring, and since the nipple has no function for most males, then why do most male mammals (including humans) have nipples, but mice, rats and horses do not? In order to answer this question, we need to look at the sexually dimorphic development of MGs and nipples in different species.

In humans, a well-defined mammary bud can be observed in both male and female fetuses at the end of the first trimester of pregnancy^6^. The nipple is usually more easily seen in newborns with varying amounts of tissue in breast and no significant sex differences^7^. Sexually dimorphic development of the human breast first begins at puberty and is mainly under the influence of estrogen^8^. Nipple development in most mammals may be similar to that in humans.

In mice, the development of MG starts at embryonic day (E) 10.5, and it begins with a single-layered ectoderm enlarging to form the mammary lines, and mammary line cells then migrate to the location of the future MG^9,10^. At E11.5, multilayered ectodermal placodes are observed. The mammary placodes form bulbs of epithelial cells and develop knob-like structures at E12-E13.5, then go into the underlying dermis at around E13.5 and mesenchymal cells around the epithelial condense and become the mammary mesenchyme^11^. At E15.5-E16.5, female MG development continues, with epithelial cell proliferation and elongation in the bud leading to the formation of a sprout that invades the fat pad precursor, and the sprout subsequently branches into the fat pad, giving rise to the rudimentary ductal tree by E18.5^12^. Nipple formation occurs through interactions between the overlying skin and the primary mammary mesenchyme and involves thickening of the epidermis and then generation of a nipple sheath from keratinocytes at the point where the primary duct connects to the skin surface^13^.

The literature about male MG development is very limited. Several publications mentioned that, in male mice, because of the effect of androgen during E13.5-E15.5, MGs regress and nipples disappear at E16.5; Sexual dimorphism in MG is achieved primarily during the embryonic stages between E14 and E16.5 in mice but later in humans^13,14^. It was suggested that in humans and most other mammals, the sexual dimorphism is accomplished via a different mechanism, and the MG is not severed during embryogenesis. Instead, male and female MGs undergo similar development until puberty when hormonal regulation specifies their size^15^.

Recently, the development of male MG drew attention because the incidence of gynecomastia and male breast cancer has increased in human populations^16^. Breast cancer is relatively rare in men compared to the high rates in women with male breast cancers accounting for less than 1% of all diagnosed cases^17^. Interestingly, male breast cancer incidence rose by 40% from 1975 to 2015, exceeding that of women by 25%^18^. The etiology of male breast cancer is complicated and not well understood. According to the dramatically increased incidence in the past decades, endocrine disrupting chemical exposure is a strongly suspected contributing factor. There is some evidence that male breast cancers are more likely to express the receptors of estrogen or androgen than female breast cancers and less likely to overexpress HER2^19,20^. Although male breast cancer is relatively rare, gynecomastia, which is defined as benign proliferation of male breast glandular tissue, is very common, and the prevalence is 50% to 60% in adolescents, and up to 70% in men aged 50 to 69 years^21^. A recent review paper summarized 16 reasons for the etiology of gynecomastia in adolescent boys, and the majority are related to increased estrogen levels^22^. Lately, several studies suggest prenatal or neonatal exposure to estrogenic chemicals induces mammary malformation in males^23–27^. These publications indicate that endocrine disrupting chemicals may induce diseases related to breast development, gynecomastia and breast cancer in later life. Unfortunately, all of these studies were performed using mice or rats. The mechanism of how prenatal and/ or neonatal androgen or estrogen affects MG development in nipple loss mammals (such as mice and rats) may be different from that in humans. The fetal testicles of all mammals produce testosterone (T) during the sexual differentiation stage; how can we explain why male mice and rats do not have nipples while most other male mammals do? The answers in current literature are focused on the timing of mammogenesis initiation and T production in fetal testes^4,28^. As MG development begins before fetal testes produce T in all reported mammals including humans and mice, how can the MG and nipple development only be affected in mice and rats, but not in others at this embryonic stage? Clearly, the existing literature can’t explain these differences.

Interestingly, male guinea pigs (*Cavia porcellus*), another rodent, are born with nipples, similar to humans and many other mammals^29^. In this study, we compared sexually dimorphic development of MG between mice and guinea pigs and revealed the mechanism behind the differing nipple development in male mice (nipple loss) and male guinea pigs (nipple retention).

## Results

### Differential development of the nipple in male mice and guinea pigs.

**Figure 1:**
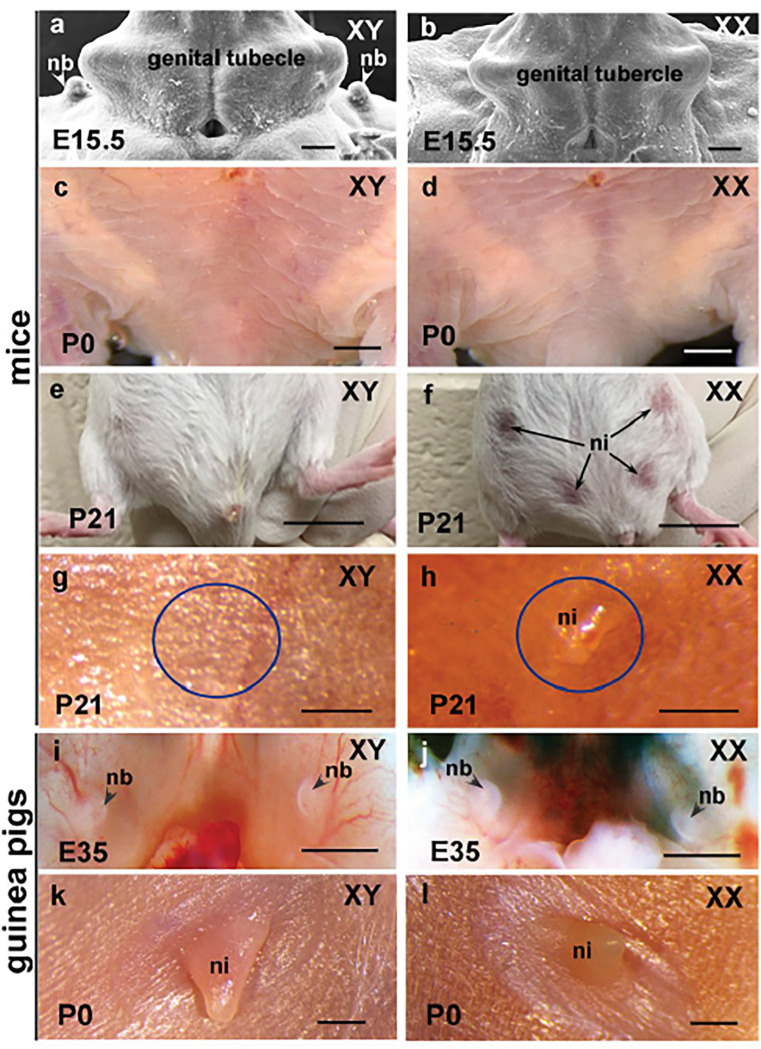
Ontogeny of mouse and guinea pig nipples. (a, b), SEM images showing sexual dimorphism of E15.5 nipple buds in mice, note that the nipple buds can be observed in males, but absent in females at this stage. (c, d), No nipples are observed in newborn male and female mice. (e-h), Ventral views of P21 mice show position (f) and morphology (h) of nipples of females. Note that male mice lost nipples during development (e, g). (i, j), At E35, both male and female guinea pigs have nipple buds with similar size and morphology. (k, l), Nipples of newborn male and female guinea pigs. Stage is indicated at bottom left of each image, and sex is indicated by chromosome symbols XY (male) and XX (female) at top right of each image in all figures. Blue circles in g and h show similar position of male and female nipple. nb, nipple bud; ni, nipple. Scale bars in a and b, 100μm; in c, d, g-l, 1mm; in e and f, 1cm.

After comparison of MG and nipple development between mice and guinea pigs, we found that in mice, differential development of male and female MGs initiates around E14.5. At E15.5, the MGs of males actually form protrusions of nipple buds, whereas in females the mammary epithelium grows downward and invaginates into the underlying mesenchyme (Fig. 1a, b and ref^30^). At later stages, the MGs continue development in females but undergo atrophy in males after E15.5. At the time of birth, no nipple can be observed in both male and female mice (Fig. 1c, d); the nipples of female mice are clearly visible at weaning (Fig. 1e–h). In guinea pigs, the outgrowth of nipple buds initiates as early as E30 and is obvious under stereoscope at E35 in both males and females (Fig. 1i, j). The nipples continuously develop, and no obvious sexual dimorphism can be observed at the time of birth (Fig. 1k, l). The results suggested that the nipple buds in male mice regress between E14.5 and E16.5, which is the key time of initial external genital sex differentiation^31^. In guinea pigs, the corresponding stage is around E28-E32^32^, and sex hormones appear to have no significant influence on guinea pig nipple development at this stage.

### Androgen receptor (AR) and estrogen receptor alpha (ERα) are located in mesenchyme of developing MGs of mice, but not in that of guinea pigs.

**Figure 2:**
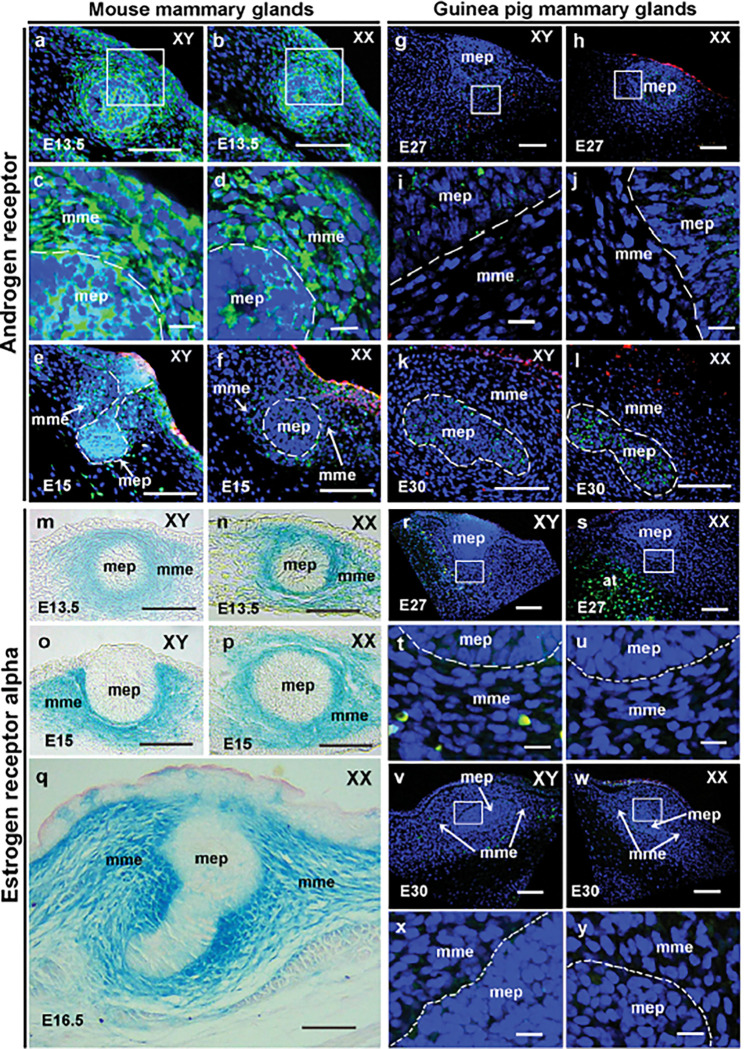
Distribution of AR and ERα in the developing mammary gland. Localization of AR (a-l) and ERα (m-y) in developing MGs of mice (a-f, m-q) and guinea pigs (g-l, r-y). (a-l and r-y) are immunofluorescence staining of AR (a-l) and ERα (r-y), AR- and ERα-positive cells are green, and blue signal is DAPI. (m-q) show Esr1 expression using Esr1cre LacZ (blue) reporter mice. Stage and sex are indicated the same as in Figure 1. White square-marked regions in a, b, g, h, r, s, v and w are shown at a higher magnification in c, d, i, j, t, u, x and y, respectively. White broken lines in (c-f, i-l, t, u, x and y) show the boundary of mammary epithelium and mesenchyme. Red color in images indicates non-specific staining. Note that both AR and ERα were not detected in the developing mammary mesenchyme of guinea pigs (g-l, r-y); E15 male mice exhibit more activated (nuclear) AR (e) than female mice (f); ERα is present in adipose tissue of female guinea pigs (s); Esr1 is strongly expressed in the mammary mesenchyme of E16.5 female mice (q), but male MG atrophies at this stage. mep, mammary epithelium; mme, mammary mesenchyme. Scale bars: a, b, e-h, k-s, v and w, 20μm; c, d, i, j, t, u, x, and y, 5μm.

The differential development of MG and nipple between mice and guinea pigs at the time of sexual differentiation suggests androgen signal may play a role in nipple loss in male mice. Both androgen production and AR activity play key roles in androgen signaling. As androgen production in fetal testes of mice and guinea pigs initiates before genital sex differentiation^33,34^, we hypothesized that AR is differentially expressed in developing MGs between mice and guinea pigs. To test this hypothesis, we performed immunohistochemistry to visualize AR protein in developing MGs of mice and guinea pigs. Our results indicated that before genital sex differentiation, AR protein was localized in cytoplasm of developing MGs in both male and female mice at E13.5 (Fig. 2a–d). Two days later, AR-positive cells in developing MGs of male mice were much more prevalent than in females. A small portion of nuclear-localized AR could be detected in mammary mesenchyme of E15.5 females (Fig. 2f). In same stage males, it’s hard to clearly distinguish AR localization because too many AR-positive cells were detected in mammary mesenchymal and epithelial cells (Fig. 2e). Compared with mice, no AR protein was detected in mammary mesenchyme of E27 or E30 male and female guinea pigs. Some positive signal was found in E30 mammary epithelium, but no nuclear AR signal was detected (Fig. 2g–l).

Estrogen has several receptors. ERα is considered the primary receptor for MG development and function^35,36^. To determine the responsiveness of the MG to estrogen signaling in mice and guinea pigs, we first investigated ERα localization in male and female mice before and during genital sexual differentiation using Esr1Cre lacZ reporter mice. The results showed that *Esr1*-positive cells were localized in mammary mesenchyme during sex differentiation, and males and females had similar *Esr1* expression patterns at these stages (Fig. 2m–p). At E16.5, the male MG diminished, while in females, mammary epithelial cells began to actively proliferate and the mammary buds sprouted down out of the dense mesenchyme, and *Esr1* was still strongly expressed in mammary mesenchymal cells surrounding the epithelium buds (Fig. 2q). The ERα protein was also detected in developing MGs of both male and female mice, highly expressed in mammary mesenchyme of E15.5 female MGs, but mainly localized in cytoplasm (Figure S1a-f). We then performed ERα protein localization in MGs of E27 and E30 guinea pigs, and no ERα-positive cells were detected in mammary mesenchyme and epithelium of both males and females (Fig. 2r–y), but multiple ERα-positive cells were detected in adipose tissue surrounding MGs especially in female guinea pigs (Fig. 2s).

### AR and ERα signaling determine differential nipple development between mice and guinea pigs.

**Figure 3:**
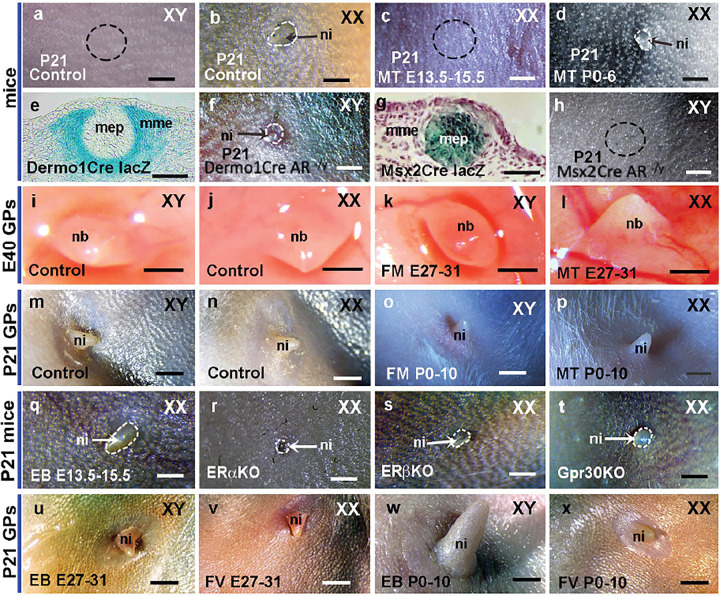
Differential effects of androgen and estrogen signaling on nipple development in mice and guinea pigs. (a-d), Nipples of P21 control male (a) and female (b), prenatally (c, n=3) and neonatally (d, n=3) MT-treated female mice. Note that prenatal, but not neonatal androgen treatment induced nipple loss in female mice. (e-h), Deletion of AR in mesenchyme (using Dermo1cre) or epithelium (using Msx2Cre) in mice. Note that male mice developed nipples after AR deletion in mammary mesenchyme (f, n=3), but no effect was observed after AR deletion from epithelium (h, n=4). (i-l), Nipple buds (NBs) of E40 control male (i) and female (j), prenatally (E27–31) flutamide-treated male (k, n=3) and MT-treated female (l, n=3) guinea pigs. (m-p), Nipples of P21 control male (m) and female (n), neonatally (P0–10) flutamide-treated male (o, n=4) and MT-treated female (p, n=4) guinea pigs. Note that prenatal and neonatal MT or flutamide treatment had no obvious effect on nipple development in guinea pigs. (q-t), Nipples of P21 prenatally (E12.5-E16.5) EB-treated female (q, n=3), ERα knockout female (r, n=3), ERβ knockout female (s, n=3) and Gpr30 knockout female (t, n=3) mice. (u-x), Nipples of P21 prenatally (E27–31) EB-treated male (u, n=4), prenatally fulvestrant-treated female (v, n=3), neonatally (P0–10) EB-treated male (w, n=4) and neonatally fulvestrant-treated female (x, n=3) guinea pigs. Note that prenatal EB treatment increased nipple size and deletion of ERα reduced nipple size in female mice; deletion of ERβ or Gpr30 had limited effect on mouse nipple development. In guinea pigs, neonatal but not prenatal EB (or fulvestrant) treatment increased (or decreased) the nipple size. Blue color in (e and g) indicates LacZ-positive cells. Black broken circles in (a, c and h) show nipple development position under specific conditions. nb and ni are the same as in Figure 1. mep and mme are the same as in Figure 2. FM, flutamide; EB, estradiol benzoate; FV, fulvestrant; GP, guinea pig; MT, methyltestosterone; Scale bars: a-d, f, h, i-l and q-t, 500μm; m-p and u-x, 2mm; e and g, 20μm.

The sexually dimorphic development of nipples in mice was thought to be caused by T at sex differentiation^28^. T is produced in the fetal testes of all mammals, including guinea pigs and mice during sexual differentiation^37^. Interestingly, only in several species, such as male mice and rats, does the MG atrophy during genital sexual differentiation. To test the differential function of androgen signaling in developing MGs of mice and guinea pigs, we first treated mice at 13.5–15.5 days of gestation with methyltestosterone (MT); female fetuses exposed to MT also showed atrophy of the nipples, similar to those of control males (Fig. 3a–c, Table 1). When exposed to MT at the neonatal stage from the day of birth to 6 days after birth (P0-P6), the females’ nipples were still present but smaller (p = 0.0262) than those of controls (Fig. 3d, Table 1). Additionally, our experiments showed that prenatal but not neonatal AR antagonist flutamide treatment induced nipple development in male mice, and prenatal and neonatal flutamide had no obvious effect (p ≥ 0.1038) on female nipple development (Table 1). The results suggested that the nipple loss of male mice was mainly controlled by prenatal androgen signaling. Dermo1cre and Msx2cre were successfully used in tissue-specific knockout of genes expressed in mesenchymal and ectodermal tissues^31,38^. To test the tissue-specific AR function in developing MG, we performed AR knockout at mammary mesenchyme and ectoderm using Dermo1cre and Msx2cre respectively. We found only AR knockout in mesenchyme (with Dermo1cre) led to nipple retention in male mice (Fig. 3e, f, Table 1), and knockout of AR in male mammary epithelium (using Msx2cre) exhibited a phenotype similar to control males (Fig. 3g, h, Table 1). All these results suggested that AR in mammary mesenchymal cells is required in male nipple loss in mice. In comparison, we also tested the effect of prenatal and neonatal androgen on nipple development in guinea pigs. Both male and female guinea pigs developed nipples, which could be visualized at embryonic stage (Fig. 1I, J). Flutamide or MT treatment at E27 to E31 had no obvious effect (p ≥ 0.0973) on nipple development in guinea pigs (Fig. 3i–l, Table 1). No sexually dimorphic differences of nipple morphology between control male and female guinea pigs were noticed at both E40 and P21 (Fig. 3i, j, m and n), and neonatal flutamide or MT treatment did not significantly affect (p ≥ 0.0831) nipple development in guinea pigs of both sexes (Fig. 3m–p, Table 1). The significant changes (p ≤ 0.0284, Table 1) of anogenital distance (AGD) in MT-treated females and flutamide-treated males suggested that the lack of effect of MT or flutamide on nipple development was tissue-specific, and the results were consistent with the outcome that few AR-positive cells were detected in developing MGs of guinea pigs (Fig. 2g–l).

We next asked whether estrogen signaling plays a role in MG development and sex differentiation and then contributes to male nipple loss in mice. Estradiol benzoate (EB, 200μg/kg) treatment from E13.5 to E15.5 did not induce nipple development but reduced AGD (p ≤ 0.0216) in male mice (Table 1) and increased (p ≤ 0.0173) the size of nipples but had no significant effect on AGD (p ≥ 0.1015) in females compared with controls (Fig. 3q, Table 1). Genetic approach was also used to knock out 3 major estrogen receptors, ERα, ERβ and GPR30. Deletion any one of these 3 receptors led to no nipple development in male mice, similar to control males (Table 1). Deletion of ERα reduced (p = 0.0074) the nipple size of P21 female mice, and deletion of ERβ or GPR30 had little effect on nipple size of P21 female mice (Fig. 3r–t, Table 1). The results suggested that estrogen receptor signal has no effect on nipple loss in male mice but can promote nipple development in female mice mainly through ERα. In guinea pigs, EB (100μg/kg) or ER antagonist fulvestrant (10mg/kg) treatment daily at E27–31 had no effect (p ≥ 0.0912) on nipple length in both males and females (Figs. 3u, v, S2a, b and Table 1), but EB treatment every day at neonatal stage (P0-P10) dramatically increased (p ≤ 0.0002) the nipple size of both P21 male and female guinea pigs (Figs. 3w, S2c and Table 1). Fulvestrant treatment every day at P0-P10 showed a slight but significant reduction in nipple size of both male (p = 0.0371) and female (p = 0.0414) guinea pigs (Figs. 3x, S2d and Table 1). The results indicated that neonatal estrogen treatment promotes nipple growth in both male and female guinea pigs.

### AR signal promotes tissue-specific cell death and cell proliferation in developing MGs of mice, but not guinea pigs at genital sex differentiation.

**Figure 4:**
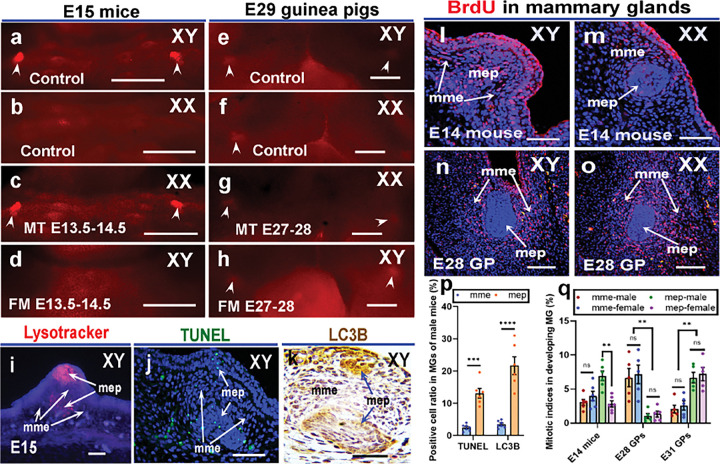
Androgen signaling controls differential cell death and proliferation in developing MGs between mice and guinea pigs. (a-d), LysoTracker Red signal shows cell death in E15 mouse MGs of control males (a, n=4), control females (b, n=4), MT-treated females (c, n=3) and flutamide-treated males (d, n=3). (e-h), LysoTracker Red signal shows cell death in E29 guinea pig MGs of control males (e, n=3), control females (f, n=3), MT-treated females (g, n=3) and flutamide-treated males (h, n=3). (i-k), Longitudinal sections (cross the body trunk) of MGs show LysoTracker Red (i, n=4,), TUNEL (j, in green, n=6) and LC3B localization (k, in brown, n=6) in E15 male mice, with epidermal skin on the top. (l-o), BrdU labeling (in red) shows cell proliferation in developing MGs of male and female mice (l and m) and guinea pigs (n and o), blue is Dapi. (p and q), TUNEL- and LC3B-positive cell ratios in E15 MGs of male mice (p, n=6), and BrdU-positive cell ratios in developing MGs of male and female mice (q, n=6) and guinea pigs (q, n=5). Data are shown with mean ± SE, **p≤0.01, ***p≤0.001, **** p≤0.0001, ns indicates no significant difference. Note that more mesenchymal cell proliferation before nipple bud formation (E28), but more epithelial cell proliferation after nipple bud formation (E31) was detected in MGs of male and female guinea pigs. White arrowheads in a, c and e-h point to nipple buds. mep and mme are the same as in Figure 2; GP, FM and MT are the same as in Figure 3. Scale bars: a-h, 500μm; i-o, 20μm.

We next asked how the male mice lose nipples. As the MGs in male mice diminish at E16.5, we hypothesized that androgen promotes cell death in developing MGs at the time of E14.5-E15.5 and plays a role in male nipple loss. To test this hypothesis, we performed cell death analysis on E15 mouse embryos using LysoTracker Red. We found the males, but not the females showed evident cell death in developing MGs (Fig. 4a, b). MT-treated female MGs showed a similar pattern of cell death to that of control males (Fig. 4c), and no LysoTracker signal was detected in MGs of flutamide-treated males (Fig. 4d). We also performed cell death analysis on E29 guinea pig embryos. Both the male and female MGs showed a similar pattern of very weak LysoTracker signals (Fig. 4e, f). MT- or flutamide-treated MGs showed a similar LysoTracker Red staining pattern to that of controls (Fig. 4g, h). The results suggested AR signal promotes cell death in developing male MGs of mice but not guinea pigs at sex differentiation. We next asked whether AR promotes programmed cell death through apoptosis or autophagy pathway. To test this, we performed TUNEL analysis and microtubule-associated protein 1 light chain 3 beta (LC3B) immunohistochemistry on E15 mammary glands of male mice and the results showed that both TUNEL- and LC3B-positive cells were found in mammary epithelium and mesenchyme (Fig. 4i–k). To quantify the TUNEL- and LC3B-positive cell numbers, we counted all positive cells and total cells in all sections of each MG of E15 male mice (n = 6). The average positive cell ratios of TUNEL and LC3B in mammary epithelium were 13.1% and 21.6% respectively, significantly higher than TUNEL- and LC3B-positive cell ratios in mammary mesenchyme, which were less than 2.5% and 3.5% respectively (p < 0.0001; Fig. 4p).

It was interesting that the mammary glands formed protrusions in both sexes of guinea pigs but only in male mice, and at the same time the programmed cell death was only found in mammary glands of male mice but not in male and female guinea pigs. These results suggested that differential cell proliferation may contribute to the sexually dimorphic development of MGs in mice. To test this hypothesis, we determined mitotic indices using BrdU-labeled cells. The results showed that in mammary mesenchyme of E14 mice, the mitotic indices had no significant difference between males and females (p = 0.2976, n = 6), but in the mammary epithelium, the mitotic indices in males (6.9%, n = 6) were much higher than in females (3.1%, p = 0.0013, n = 6; Fig. 4l, m, q). In E28 guinea pigs, much more BrdU-positive cells were found in mammary mesenchyme (male, 6.68%; female, 7.36%) than in mammary epithelium (male, 1.04%, p = 0.0036, n = 5; female, 1.34%, p = 0.0038, n = 5) and no sex differences (p ≥ 0.1315) of cell proliferation were detected (Fig. 4n, o, q). In E31 guinea pigs, mammary protrusions can be detected, and more BrdU-positive cells were found in mammary epithelium (male, 6.86%; female,7.22%) than in mesenchyme (male, 2.06%, p = 0.0014, n = 5; female, 2.09%, p = 0.0046, n = 5) and no sex differences (p ≥ 0.4578) were detected (Fig. 4q).

### Transcripts of Ar and target genes were detected in the MGs of male mice but not guinea pigs during sex differentiation.

**Figure 5:**
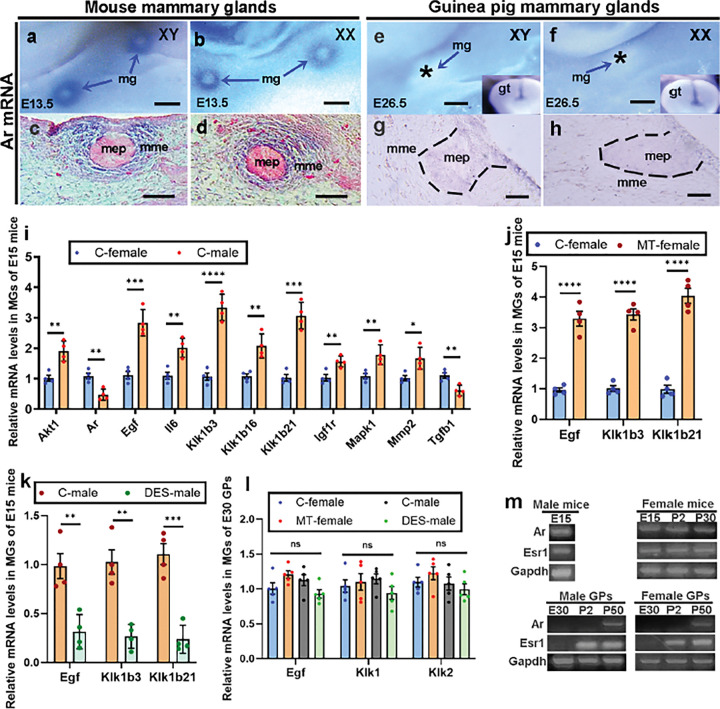
Temporal and spatial Ar mRNA expression determines differential development of nipples between mice and guinea pigs. (a and b), Ar mRNA localization in MGs of E13.5 male (a) and female (b) mice. (c and d), Sections of MGs in a (c) and b (d) show tissue-specific Ar mRNA expression (blue), red is counter staining with eosin. (e-h), Ar mRNA localization in MGs of E26.5 guinea pigs using whole mount (e and f) and section (g and h) in situ hybridization, with the small images in the bottom right of e and f showing genital tubercle from the same embryo for comparison. Note that Ar mRNA was detected in developing mammary mesenchyme of mice, but not guinea pigs. (i-l), Relative mRNA expression levels of androgen-responsive genes in E15 MGs of control male and female (i), MT-treated female (j) and DES-treated male mice (k) and guinea pigs (l). Data are shown as mean ± SE of relative fold changes, n=4 for mice and n=5 for guinea pigs. Note that selected androgen-responsive genes (Egf, Klk1b3 and Klk1b21) showed increased mRNA levels in MGs of male and MT-treated female mice only, but not guinea pigs. (m), Ar and Esr1 cDNA were detected in MGs of different stages (prenatal, neonatal and pubertal) of mice and guinea pigs. Note that Ar and Esr1 were not detected in MGs of prenatal (E30) male and female guinea pigs, and Ar was also not detected in neonatal MGs of guinea pigs. *p≤0.05, ** p≤0.01, *** p≤0.001, **** p≤0.0001. ns indicates no significant difference. mg, mammary gland; gt, genital tubercle; GP is the same as in Figure 3; mep and mme are the same as in Figure 2. Scale bars: a, b, e, and f, 200μm; c, d, g and h, 20μm.

Our findings showed that AR protein was localized in MGs of mice but not guinea pigs during sex differentiation, which suggested that *Ar* transcription might be activated in mice, but not in guinea pigs. To test this, we performed *in situ* hybridization on male and female E13.5 mice and E26.5 guinea pigs. The results showed that *Ar* mRNA expression patterns in MGs of male and female mice were the same, being strongly expressed in mammary mesenchymal cells (Fig. 5a–d). In E26.5 guinea pigs, no *Ar* mRNA was detected in MGs of either males or females, but positive *Ar* mRNA was detected in the genital tubercles of the same embryos (Fig. 5e–h). AR activation was mainly determined by measuring the expression levels of downstream AR-target genes^39^. To reveal AR downstream genes in developing MGs at sex differentiation, we compared the AR signaling gene expression (quantitative RT-PCR) between E15 male and female MGs using Androgen Receptor Nuclear Signaling PCR array (Bio-rad, gene list can be found on https://commerce.bio-rad.com/en-cn/prime-pcr-assays/pathway/transcription-androgen-receptor-nuclear-signaling). We found 11 out of the total 43 AR signaling pathway genes showed significant (p ≤ 0.05; n = 4) fold change (greater than 1.6-fold; Fig. 5i). These 11 genes (*Akt1*, *Ar*, *Egf*, *Il6*, *Klk1b3, Klk1b16, Klk1b21, Igf1r, Mapk1, Mmp2* and *Tgfb1*) were designated as androgen-responsive genes during mouse MG development at sex differentiation. *Ar* and *Tgfb1* showed more than 2-fold downregulation in males. *Egf, Klk1b3 and Klk1b21* showed more than 2.8-fold upregulation (p ≤ 0.0004) in males and the three upregulated genes were selected to compare the AR nuclear signaling between mice and guinea pigs. Compared with E15 female mice, these 3 genes were significantly upregulated in MT-treated females (p < 0.0001 for all 3 genes, n = 4; Fig. 5j). All three genes were significantly downregulated in diethylstilbestrol (DES)-treated males compared with control males (*Egf*, p = 0.0049; *Klk1b3*, p *=* 0.0015; *Klk1b21*, p = 0.0005; n = 4; Fig. 5k). Because we could not find Klk1b3 and Klk1b21 in the available guinea pig gene list, we performed a protein blast search and found that both genes are highly conserved with kallikrein A1 (also known as Klk1) and kallikrein 2 (also known as Klk2). We then compared mRNA levels of *Egf*, *Klk1* and *Klk2* genes among E30 control female and male, MT-treated female, and DES-treated male guinea pigs, and none of them showed significant upregulation or downregulation (p ≥ 0.0807; n = 5) (Fig. 5l). These results suggested that the AR nuclear signaling pathway is inactivated in developing MGs of guinea pigs but activated in mice.

Our experiments showed different effects of neonatal androgen and estrogen treatment on guinea pig nipple development (Fig. 3p and w). Multiple studies revealed AR was expressed in normal human breast and breast cancer tissue^40,41^. These results suggested that AR and /or ERα may be expressed at (a) specific time(s) during MG development. To test this, we performed RT-PCR to reveal *Ar* and *Esr1* mRNA expression in MGs of guinea pigs and mice at prenatal, neonatal and pubertal stages and the results were shown in Fig. 5m. We can see that the transcripts of *Ar* and *Esr1* were detected in MGs of E15 male and female and P2 and P30 female mice. Interestingly, in both sexes of guinea pigs, neither *Ar* nor *Esr1* mRNA was detected in embryonic (E30) MGs, *Esr1* mRNA was detected in neonatal (P2) and pubertal (P50) MGs, but *Ar* mRNA was detected only in pubertal (P50) MGs.

### Estrogen antagonizes androgen signaling, regulates differential expression of Wnt pathway genes, and then induces prenatal nipple bud formation in male and female mice.

**Figure 6:**
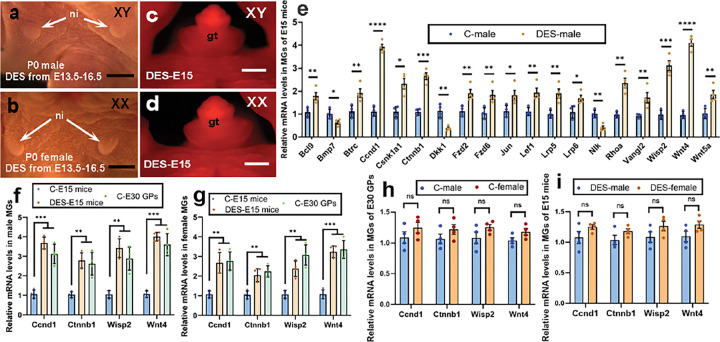
Prenatal estrogen treatment induces nipple formation in male mice through activation of Wnt signaling pathway genes. (a, b), The nipples of male (a) and female (b) newborn mice that received DES prenatally are visible. Note that no nipples were detected in newborn mice that did not receive DES. (Figure 1c and d). (c, d), LysoTracker Red signal was not detected in E15 MGs of DES-treated (E13.5 and E14.5 once daily) male (c, n=3) and female (d, n=3) mice. Note that the signal was detected in MGs of control male and MT-treated female mice (Figure 4a, c). (e-i), Relative mRNA expression of Wnt pathway genes in MGs of mice and guinea pigs at sex differentiation. (e), Relative mRNA levels in MGs of DES-treated and control E15 male mice. (f, g), Relative mRNA levels in MGs of DES-treated and control male and female mice and guinea pigs. (h, i), Relative mRNA levels in MGs of control guinea pigs (h) and DES-treated mice (i). Note that the expression levels of Wnt signal genes in MGs of control E30 guinea pigs (E30 male GPs) were similar to those in DES-treated E15 mice (f, g). No sexually dimorphic expression of selected Wnt pathway genes was detected in MGs of control E30 guinea pigs (h) and DES-treated E15 mice (i). Data are shown as mean ± SE, n=4. *p≤0.05, ** p≤0.01, *** p≤0.001, **** p≤0.0001. ns indicates no significant difference. ni is the same as in Figure 1, gt is the same as in Figure 5. Scale bars: a and b, 1mm; c and d, 500μm.

In most mammals, including humans and guinea pigs, nipple bud development begins at the embryonic stage, and nipples can be visibly observed at the time of birth^42^. Mice and rats, regardless of sex, do not have visible nipples at birth (Fig. 1c, d and ref^43^). Our results showed that MGs of male mice and MT-treated female mice underwent extensive cell death and estrogen treatment reduced the expression of androgen-responsive genes in MGs of male mice, suggesting that estrogen treatment can antagonize androgen signaling in MG development. To test the effect of estrogen treatment on MG and nipple development in mice, we treated the pregnant dams with different dosages of DES from E13.5-E16.5 and found when we increased the DES dosage to 200μg/kg, both male and female mice showed visible nipples at birth (Fig. 6a, b). In addition, no programmed cell death (LysoTracker Red-positive signal) was detected in DES (at E13.5 and E14.5)-treated E15 male and females MGs (Fig. 6c, d). The result suggested that high-dose DES treatment induces nipple development at embryonic stage in both male and female mice.

Wnt signaling plays an important role in MG development. Wnt pathway genes are involved in the early development of the MG in embryos and their role extends to postnatal differentiation^44^. To reveal the genes involved in MG and nipple development after DES treatment, we compared the expression levels of Wnt signaling genes in MGs of E15 control males and DES-treated males using Mouse WNT Signaling Pathway PCR array (Qiagen PAMM-043Z). We found 19 out of the total 84 Wnt signaling genes showed significant (p ≤ 0.05; n = 4) fold change (greater than 1.6-fold), 16 genes (*Bcl9*, *Btrc, Ccnd1, Csnk1a1, Ctnnb1, Fzd2, Fzd6, Jun, Lef1, Lrp5, Lrp6, Rhoa, Vangl2, Wisp2, Wnt4* and *Wnt5a*) were upregulated in DES-treated males, and 3 of them (*Bmp7, Dkk1* and *Nlk*) were downregulated (Fig. 6e). DES treatment at the sex differentiation stage induced nipple bud development in mice and gave rise to visible nipples at birth, similar to the observations in control guinea pigs. We hypothesized that similar mechanisms may be involved in nipple bud development in DES-treated mice and control guinea pigs. To test this hypothesis, we selected 4 genes (*Ccnd1, Ctnnb1, Wisp2* and *Wnt4*) that were upregulated more than 2.5-fold from the 19 differentially expressed genes found in the comparison of DES-treated male mice with control male mice and compared their expression levels between mice and guinea pigs. Compared to MGs of E15 control male mice, the mRNA levels of *Ccnd1, Ctnnb1, Wisp2* and *Wnt4* in MGs of E30 control male guinea pigs increased by 3.09-, 2.78-, 2.84- and 3.65-fold, respectively (p-values were 0.0003, 0.0019, 0.0014 and 0.0001 respectively; n = 4), and the relative expression levels of the 4 genes in MGs of control guinea pig males were similar to those in DES-treated male mice (p ≥ 0.1024; Fig. 6f). In addition, the expression levels of these four genes in control E30 female guinea pig MGs were similar to those in DES-treated female mice (p ≥ 0.0778; n = 4), and were significantly increased (p ≤ 0.0020; n = 4) compared with control female mice (Fig. 6g). Surprisingly, no significant sex differences in gene expression were found in control guinea pigs (p ≥ 0.0936; Fig. 6h) and high-dose DES-treated mice (p ≥ 0.1147; Fig. 6i).

## Discussion

**Figure 7:**
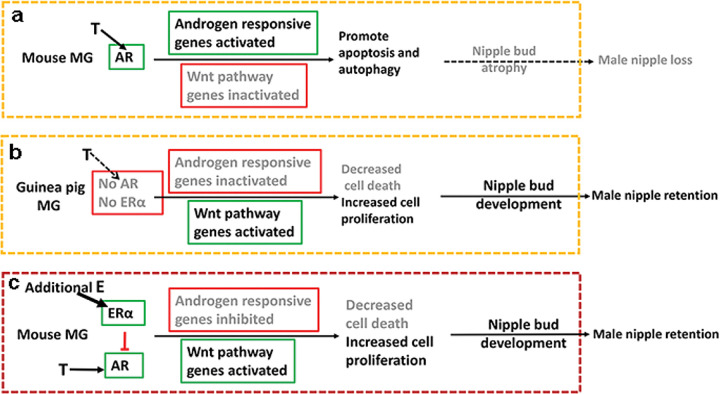
Tissue-specific AR and ERa signaling during genital sexual differentiation determine the loss or retention of male nipples in mammals. Active signaling pathways are shown in black text; diminished signaling pathways are shown in gray text. Green boxes indicate increased activity, and red boxes indicate decreased activity. (A) In male mice, higher androgen (T) levels activate AR during the prenatal sexual differentiation window and upregulate androgen nuclear signal pathway genes and promote apoptosis and autophagy mainly in mammary epithelial cells, leading to nipple loss in male mice. (B) In male guinea pigs, high levels of androgen (T) are also produced in fetal testes during sexual differentiation, but since no AR is expressed in developing MGs at this stage, androgen nuclear signal pathway genes are inactivated, Wnt pathway genes are upregulated, increasing mammary epithelial cell proliferation and decreasing cell death, inducing nipple formation, so nipple remains after birth. (C) AR and ERα both are expressed at sexual differentiation stage. When male and female mice are exposed to high dose of estrogen (E), ER signal antagonizes AR signal, androgen nuclear signal pathway genes are downregulated, Wnt pathway genes are upregulated, decreasing cell death and increasing cell proliferation, inducing nipple formation in both male and female mice at prenatal stage, leading to visible nipples at birth.

Our results demonstrated that AR mRNA and protein are detected in embryonic MGs of mice but not guinea pigs, which is the main reason for the loss of nipples in male mice. On the other hand, male guinea pigs develop nipples due to the tissue-specific loss of AR transcription during embryonic development. The testicles of male mouse fetuses produce T during sexual differentiation. T activates AR, induces the expression of AR nuclear signaling pathway genes, promotes apoptosis and autophagy, inhibits the expression of Wnt pathway genes (*Wnt4*, Wnt5a, *Wisp2*, *Ctnnb1* etc.), and then leads to nipple loss (Fig. 7a). The testicles of male guinea pig fetuses also produce T during sexual differentiation. Since there are no AR mRNA and protein in the developing guinea pig MGs, the AR nuclear signaling pathway genes are inactivated and cell death is diminished, the Wnt pathway genes are activated and induce nipple bud development, leading to visible nipples at birth (Fig. 7b). When male mouse fetuses are exposed to additional high doses of estrogen, ERα is activated in the developing MG, and ERα signaling antagonize AR signal, inhibits the expression of AR nuclear signaling pathway genes and promotes the expression of Wnt pathway genes, thereby inducing nipple bud development and leading to visible nipples at birth (Fig. 7c). Prenatal exposure of guinea pig fetuses to estrogen has no significant effect on nipple development in either males or females due to the lack of *Ar* and *Esr1* expression. The cellular mechanism by which estrogen induces nipple retention in male mice may be similar to that in control guinea pigs. The control female mice also do not have visible nipples at birth, but female mice prenatally treated with estrogen develop visible nipples at birth, indicating that androgen signaling during sexual differentiation affects nipple formation not only in male mice but also in female mice.

The development of female MG has been well studied in animal models (mostly mouse) and humans^45,46^. The literature on male MG development is limited, and the limited studies have focused primarily on mice and rats^24,26,47^. Studies of endocrine control of MG development in other mammals are mainly focused on puberty and later lactation stages^48–50^. Our comparative study between mice and guinea pigs revealed that the major sexually dimorphic development of MG was found in embryonic mice at the sexual differentiation stage, but not in embryonic guinea pigs. The main reason for this difference is because *Ar* transcription was found in developing MGs of mice but not in guinea pigs. The effect of androgen signaling on MG regression was reported 40 years ago^51,52^. Using *Tfm* mice and organ culture, Drews^51^ found that T induced degeneration of the MG via the mesenchyme. The same year, Kratochwil^52^ found male MGs were responsive to androgen only at embryonic days 13–15. The results of tissue-specific deletion (using Dermo1cre and Msx2cre) of AR and time-specific MT treatment (prenatal E13.5–15.5 and neonatal P0–6) in this study clearly revealed tissue- and time-specific AR function in nipple loss of male mice. Using a genetic approach, our results confirmed that only mesenchymal AR causes MG atrophy and nipple loss in male mice and delays nipple formation in females. Activation of AR at the prenatal sex differentiation stage is required to promote cell death in MGs. In guinea pigs, AR mRNA and protein are not detected in prenatal sex differentiation and neonatal stages, and treatment with MT and AR antagonist during both the stages had no obvious effect on nipple development. Clearly, the differential nipple development between male mice and guinea pigs is mainly determined by temporal and spatial *Ar* expression in developing MGs at the time of sexual differentiation. The question still remains how *Ar* transcription is activated in MGs of mice but inactivated in those of guinea pigs. The regulation of *Ar* transcription is complicated, and multiple mechanisms have been reported. Mclean et al^53^ found human-specific loss of regulatory DNA of *Ar* gene induced penile spine loss; DNA methylation or histone modification could regulate *Ar* gene transcription and was revealed in the prostate^54,55^; interestingly, Dunbar et al^56^ found that parathyroid hormone-related peptide (Pthrp / Pthlh) was required for sexual dimorphism during embryonic mammary development in mice. We performed RT-PCR to detect the *Pthlh* and its receptor *Pth1r* mRNA and found they were expressed in MGs of E30 and newborn guinea pigs of both sexes (Figure S3). The results suggested that PTHrP may affect AR signaling in developing MGs of mice but may not contribute to the *Ar* transcription inactivation in developing MGs of guinea pigs. Obviously, epigenetic regulation, specific enhancers, noncoding RNA, microRNA and even indirect effects may be possible reasons. Therefore, the deep mechanism of tissue-specific *Ar* transcriptional activation in different species needs further study.

Recently, several studies suggested prenatal or neonatal exposure of estrogenic chemicals induces mammary malformation of males^23–27^. Unfortunately, none of these studies were performed using male-nippled animals. Consistently, our data suggested that both AR and ERα mRNA and protein are expressed in developing MGs of mice during embryonic stage, and prenatally DES-treated male mice form visible nipples at birth. Our studies on genetic knockouts of different estrogen receptors demonstrated that estrogen induces nipple development mainly through ERα. The results of *Esr1* expression and estrogen treatment experiments suggested that ERα signaling is activated in embryonic MGs of mice but inactivated in guinea pig MGs at the embryonic sexual differentiation stage. The mechanism underlying the effect of estrogenic chemicals in male-nippled mammals (such as guinea pigs and humans) may be different from in mice or rats. In addition, there is no evidence that AR and ERα mRNA and protein exist in developing MGs of human fetuses. Whether guinea pigs are unique or similar to the majority of other male-nippled mammals including humans needs further study.

Why do men have nipples? This is an interesting question raised hundreds of years ago, at least it intrigued Erasmus Darwin, who is Charles Darwin’s grandfather, and maybe many others before and after him^57^. This is a complicated evolutionary question and hard to have an experimentally testable answer. The more specific question is why most male mammals have nipples while male mice and rats don’t. To this question, the answers in current literature have been focused on the timing of mammogenesis initiation and fetal testicular T production. Pokharel et al ^4^ concluded that the time between the onset of MG development and the production of T is shorter in male mice than in humans, and then in mice, T induces separation of the mammary epithelium from the overlying epidermis, thereby preventing the epidermis from forming nipples. Vandenberg et al^28^ also described that the T surge induced a detachment of the MG from the epidermis, which caused nipple loss in males. In guinea pigs, MG development is initiated before testes start to produce T, and nipple buds in guinea pig embryos can be observed at the time of E30, which is the same time as external genital sex differentiation^32^. In human fetuses, T production is initiated at approximately 8 weeks of gestation, and the maximal T content in fetal testes is achieved between 10 and 15 weeks of fetal life^58^. The MG development in human fetuses starts around gestation day 28 and the nipple buds can be seen at the end of first trimester^6^. Apparently, MG development in both humans and guinea pigs initiates before fetal testicular T production begins, and nipple buds are visible during sexual differentiation, a stage at which T production peaks in most mammals. This suggests that the timing of MG development and fetal T production in humans and guinea pigs is similar to that in mice. Thus, the differential development of male nipples between mice and guinea pigs (and maybe most other mammals and humans as well) is caused by temporal and spatial activation of AR, but not the timing of mammogenesis initiation and T production in fetal testes.

Most male mammals possess nipples, with only a few species losing them during fetal development. In other words, the presence of nipples in male mammals is the evolutionary norm, while the loss of nipples during embryonic development in a few male species is the exception. In most mammals, including humans, AR is likely inactivated in the developing MG. During evolution, AR may have been ectopically expressed in the fetal MG of a few mammals, such as mice and rats.

## Materials and Methods

### Animals, anogenital distance and nipple length measurement.

*ARflox* mice were kindly provided by Guido Verhoeven (Katholieke Universiteit Leuven) via Marvin Maestrich and Connie Wang (University of Texas M. D. Anderson Cancer Center). *Dermo1Cre*, *Msx2Cre* and *ER*α, *Erβ, Gpr30* knockout mice were purchased from the Jackson Laboratory. Mice were housed in a specific pathogen-free barrier facility on 12-h light/dark cycles with access to food and water ad libitum, and all experiments were conducted in accordance with Institutional Animal Care and Use Committee protocols of University of Florida (201203399) and Southern Illinois University Carbondale (mouse, 20 – 018 and guinea pig, 20 – 014). The morning on which the vaginal copulatory plug was detected was designated as E0.5. MT treatment for prenatal and neonatal mice was performed as previously^31^. Hormone and receptor agonist and antagonist treatments in guinea pigs were performed according to the modified method as described previously^32^. In brief, flutamide (50mg/kg for guinea pigs and 120mg/kg for mice), fulvestrant (10mg/kg for guinea pigs), MT (1mg/kg for guinea pigs and 2mg/kg for mice), EB (100μg/kg for guinea pigs and 200μg/kg for mice) and DES (200μg/kg for mice) (Table 1) were dissolved in ethanol and diluted in corn oil. Treatments and corn oil vehicle controls [corn oil with 2% (vol/vol) ethanol] were administered by oral gavage (flutamide and fulvestrant) or i.p. injection (MT, EB or DES) to pregnant females and neonates. The effect of treatment on embryos was validated by measurements of AGD (Table 1). Each treatment was performed on 3 litters (n = 3). AGD and nipple length were measured under stereoscope using a caliper. Mouse tail DNA was collected for genotyping of sex using SMCX/SMCY primers^59^. Guinea pig ear DNA was extracted to reveal genetic sex using PCR amplification according to published method^60^. Sex of the fetuses of E28 or older embryos was also identified by inspection of sex-specific gonadal morphology under the stereoscope.

### Scanning Electron Microscopy (SEM), Histology and X-gal staining

SEM was performed following the previously published methods^61^. To detect β-galactosidase activity of Esr1Cre, Dermo1Cre and Msx2Cre R26R reporter mice, mouse embryos were harvested in PBS and fixed overnight in 0.2% PFA at 4°C, then stained with X-gal overnight at room temperature and fixed with 4% PFA overnight. The post-fixed samples were processed through histology protocol and embedded in paraffin wax. Sections were cut at 6–8μm sections and counterstained with hematoxylin or eosin for lacZ-stained samples^62^.

### Cell Proliferation and Cell Death.

For cell proliferation analysis, BrdU (50mg/kg) was injected to pregnant mothers at E14 in mice and E28 and E31 for guinea pigs, and embryos were collected 4h later for immunofluorescence. LysoTracker^®^ Red DND-99(Life Technologies, L7528) staining was used to detect programmed cell death in MGs of E15 mice and E29 guinea pigs following the established methods^31,62^. For quantification, all positive and total cells in the mammary mesenchyme and epithelium in all sections throughout the MG were counted using ImageJ.JS software (version 1).

### Immunofluorescence and Immunohistochemistry

The immunofluorescence of AR, ERα, BrdU and TUNEL were detected using our published methods^31,62^. The immunohistochemistry of LC3B protein was detected using ABC kit (Vector Laboratories, PK-6101) following the operation manual with rabbit polyclonal anti-LC3B IgG (ABclonal Cat# A7198, RRID:AB_2863546). Imaging was performed using a Leica DM5500 confocal microscope and a Leica DM750 microscope (Leica microsystems Inc, Buffalo Grove, IL). For quantification of mitotic indices, all BrdU-positive and total cells in the mammary mesenchyme and epithelium in all sections throughout the MG were counted using ImageJ.JS software (version 1).

#### In Situ Hybridization.

RNA whole-mount *in situ* hybridization for mice and guinea pigs was performed as described^31,62^ with modifications. In brief, to make the mouse and guinea pig RNA probes, *Ar* cDNAs were PCR-amplified (primers in supporting table) from penile tissue cDNAs of an E16 mouse and an E30 guinea pig and the products were cloned to a TA vector using InsTAclone PCR cloning kit (Thermo Scientific, K1214). After coloring, and photography under the stereoscope (Leica), the fixed MGs were embedded in paraffin wax. 8μm sections were cut and counterstained with eosin. Section in situ hybridization of *Ar* in developing MG of guinea pig was performed according to the previously described method^63^ with modifications in probe preparation and image capture (the same as immunohistochemistry).

#### RT-PCR and Quantitative PCR gene expression analysis.

Total RNA was extracted from MGs of E15, P2, P30 mice and E30, P2, P50 guinea pigs using the TRIzol method according to the operation manual (Invitrogen, Cat: 10296010, Waltham, MA, USA). RNA quality was assessed following the published method^64^. The cDNA was synthesized from 500 ng total RNA using iScript Reverse Transcription Supermix (Bio-rad, Hercules, CA, USA). PCR primers of mouse (RT-PCR only) and guinea pig (RT-PCR and qPCR) genes were designed using PrimerQuest Tool (Integrated DNA Technologies, Inc) to amplify cDNAs of around 90–150 bp (qPCR) and 500–1000bp (RT-PCR) sequences and all exhibited similar amplification efficiency (r ≥ 97) as assessed by the amplification of control cDNA dilution series. RT-PCR results were visualized using agarose gel electrophoresis followed by ethidium bromide staining. QPCR primers for mouse genes were designed and validated by OriGene. Primer sequences were summarized in supporting table. Quantitative PCR was performed using a CFX96 Real-Time PCR Detection System (Bio-rad) with iQ SYBR Green Supermix (Bio-rad) as the detector. The Real-Time QPCR was programmed the same as we recently reported^65^. Data were normalized against a housekeeping gene glyceraldehyde 3-phosphate dehydrogenase (*Gapdh*) using ΔΔCt method^66^.

### Statistical analysis.

Comparisons between two groups were performed using a two-tailed nonparametric t-test. Comparisons between multiple groups were performed using one-way analysis of variance (ANOVA) with a Turkey-Kramer post hoc test. The mitotic index was analyzed using a two-way ANOVA. Quantitative data in all graphs and tables are presented as the mean ± standard error (SE). All analyses were performed using GraphPad Prism 10 software. A p-value ≤ 0.05 was considered statistically significant.

## Supplementary Material

Supplementary Files

This is a list of supplementary files associated with this preprint. Click to download.

• Supportingmaterials.pdf

## Figures and Tables

**Table 1:** The effect of AR and ER agonist, antagonist, and genetic knockout of AR and several ERs on nipple development

Animal	treatment	P21 male		P21 female	
		Nipple length (mm)	AGD (mm)	Nipple length (mm)	AGD (mm)
mice	Control (Corn oil)	n/a	11.26 ± 1.35	0.42 ± 0.17	7.24 ± 0.93
	Prenatal MT 2mg/kg	n/a	10.92 ± 1.07	n/a	**9.76 ± 0.52** **
	Neonatal MT 2mg/kg	n/a	11.05 ± 0.77	**0.26 ± 0.08** *	**8.86 ± 0.49** **
	Prenatal FM 120mg/kg	**0.21 ± 0.06**	**8.46 ± 0.58** **	0.44 ± 0.11	7.32 ± 0.64
	Neonatal FM 120mg/kg	n/a	**9.41 ± 0.32** *	0.41 ± 0.09	7.09 ± 0.47
	Prenatal EB 200μg/kg	n/a	**9.75 ± 0.69** *	**0.66 ± 0.05** **	7.17 ± 0.63
	Neonatal EB 200μg/kg	n/a	**8.89 ± 0.47** **	**0.83 ± 0.12** **	7.02 ± 0.55
	DermolCre AR^-/y^	**0.28 ± 0.07**	**8.31 ± 0.58** ***	0.40 ± 0.06^#^	7.22 ± 0.46^#^
	Msx2Cre AR^-/y^	n/a	10.96 ± 1.04	0.38 ± 0.04^#^	7.26 ± 0.66^#^
	ERαKO	n/a	11.36 ± 0.68	**0.15 ± 0.05** **	7.13 ± 0.74
	ERβKO	n/a	11.05 ± 0.81	0.35 ± 0.07	7.32 ± 0.51
	Gpr30KO	n/a	11.17 ± 0.76	0.38 ± 0.06	7.20 ± 0.88
guinea pigs	Control (Corn oil)	2.03 ± 0.13	13.13 ± 1.24	2.05 ± 0.16	6.12 ± 0.84
	Prenatal MT 1mg/kg	1.98 ± 0.08	13.27 ± 0.95	2.06 ± 0.09	**9.36 ± 0.78** **
	Neonatal MT 1mg/kg	2.02 ± 0.11	13.57 ± 0.76	2.10 ± 0.13	**7.74 ± 0.72** *
	Prenatal FM 50mg/kg	2.05 ± 0.06	**9.16 ± 0.74** **	1.96 ± 0.11	6.25 ± 0.59
	Neonatal FM 50mg/kg	2.04 ± 0.08	**10.04 ± 0.82** **	2.13 ± 0.07	6.37 ± 0.64
	Prenatal EB 100μg/kg	2.07 ± 0.05	**10.83 ± 1.56** *	2.08 ± 0.09	6.08 ± 0.62
	Neonatal EB 100μg/kg	**6.01 ± 0.17** ***	**9.75 ± 0.79** ***	**6.11 ± 0.15** ***	5.94 ± 0.77
	Prenatal FV 10mg/kg	2.01 ± 0.12	13.29 ± 1.16	1.95 ± 0.10	5.91 ± 0.68
	Neonatal FV 10mg/kg	**1.57 ± 0.08** *	13.02 ± 1.35	**1.62 ± 0.14** *	6.19 ± 0.81

# Heterozygous females. Comparisons were made between male and female treatment and control groups.

* p ≤ 0.05,

** p ≤ 0 .01,

*** p ≤ 0.001. n = 3 litters for all treatments. Treatment stage: prenatal (mice, E13.5–15.5; guinea pigs, E27–31); neonatal (mice, P0–6, guinea pigs, P0–10). n/a, not available; AGD, anogenital distance; MT, methyltestosterone; FM, flutamide; EB, estradiol benzoate; FV, fulvestrant.

## Data Availability

All data are available upon reasonable request to the corresponding author.
